# Detection of an amplification bias associated to Leuconostocaceae family with a universal primer routinely used for monitoring microbial community structures within food products

**DOI:** 10.1186/s13104-018-3908-2

**Published:** 2018-11-08

**Authors:** Simon Poirier, Olivier Rué, Gwendoline Coeuret, Marie-Christine Champomier-Vergès, Valentin Loux, Stéphane Chaillou

**Affiliations:** 1 MICALIS, INRA AgroParisTech, Université Paris-Saclay, Domaine de Vilvert, 78350 Jouy-en-Josas, France; 2 MaIAGE, INRA, Université Paris-Saclay, Domaine de Vilvert, 78350 Jouy-en-Josas, France

**Keywords:** V3 universal primer, 16S rDNA amplification bias, MiSeq pair-end sequencing, Food microbiota, *Leuconostoc* genus

## Abstract

**Objectives:**

Sequencing of 16S rDNA V3–V4 region is widely applied for food community profiling. However, two different universal forward primers (named here MUYZER-primer1 and KLINDWORTH-primer2) targeting an identical conservative sequence upstream of the V3 region of 16S rRNA gene, and only distinguished by a single mismatch are both used. This study was carried out to compare whether the accuracy of food microbiota analysis would depend on the choice of one of these two primers.

**Results:**

Alignment of both primers with common food-borne bacteria 16S sequences revealed that the mismatch between both primers might specifically affect the amplification of *Leuconostoc, Oenococcus* and *Fructobacillus* species but not *Weissella* species. Food products containing either *Leuconostoc* and/or *Weissella* were selected for a detection test. As expected from our in silico analysis, our study showed that this mismatch induced a strong biased amplification specifically associated to the OTUs belonging to the genus *Leuconostoc* but not to the genus *Weissella*. In presence of Muyzer-primer1, none of the sequences expected for *Leuconostoc* genus was detected whereas those sequences were correctly amplified with Klindworth-primer2. Since *Leuconostoc* is an important genus in food, agro-environments and in digestive tract of animals, we recommend that Muyzer-primer1 should thus be abandoned for the bacterial characterization of their associated microbiota.

## Introduction

Bacterial 16S ribosomal DNA (rDNA) is widely used to characterize bacterial communities associated with a wide range of ecosystems. For this purpose, primers targeting conservative regions of the 16S rRNA gene are commonly designed and used to sequence hypervariable regions, which are informative for taxonomic assignment. Thus, the most critical step for accurate 16S rDNA amplicon analysis is the choice of primers. Using suboptimal primer pairs can induce serious amplification biases such as under-representation or selection against single species or even whole groups [1].

Among the hypervariable regions of 16S rDNA used for bacterial community profiling, V3–V4 has come into favor with the emergence of MiSeq pair-end sequencing technology because of its short length (~ 450 bp) which promotes improved merging of forward and reverse reads.

One of the most regularly cited forward primer targeting the V3 region was proposed by Muyzer et al. [2] in 1993. However, a more recent study conducted by Klindworth et al. [3] in 2013 and based on an in silico analysis of bacterial genomes, suggested the use of another similar primer, targeting the same region and only differentiated by a degenerated position (W) five bases downstream of the 3′ end of the primer. This degenerated position could overcome a possible bias due to a mismatch in the 16S rDNA encoding gene of some bacterial phyla. Nevertheless, the former primer proposed by Muyzer and colleagues is still routinely used to monitor many microbial communities and, in particular food microbiota [4–7].

In this study, we compared the performance of both primers using several types of food products specifically chosen because their microbiota were composed of *Leuconostoc* species, a genus which revealed to be carrying the mismatch described above in their 16S rDNA.

## Main text

### Materials and methods

Three different food products [ground beef burgers (GB), pork sausages (PS) and poultry sausages (CS)], packaged under modified atmosphere, were selected for this study. Two mock communities (MC) containing notably one strain *of Leuconostoc gelidum* subsp. *gelidum* (DSM5578) and one strain of *Weissella viridescens* (MFPC16A28-05) were also used as control as described in Poirier et al. [8]. For each food item, three batches (biological replicates) were purchased in supermarkets and stored at 8 °C until the product’s use-by date. The preparation of the bacterial pellet is detailed in Poirier et el. [8]. They were subsequently washed in 1 ml of sterile ultrapure water and collected after centrifugation at 3000×*g* for 5 min at 4 °C. DNA was then extracted using the PowerFood™ Microbial DNA Isolation kit (MoBio Laboratories Inc., Carlsbad, USA) according to the manufacturer’s instructions.

Two distinct amplifications of the ~ 450-bp V3–V4 hypervariable regions of the bacterial 16S rRNA gene were performed in parallel: one with the primer V3F (5′-ACGGGAGGC**A**GCAGT-3′) (MUYZER-Primer1) according to Muyzer et al., [2] and the second one with the primer V3F (5′-ACGGRAGGC**W**GCAGT-3′) (KLINDWORTH-Primer2) according to Klindworth et al., [3]. The same V4R primer (5′-TACCAGGGTATCTAATCCT-3′) was used for both amplifications. Forward and reverse primers carried the Illumina 5′-CTTTCCCTACACGACGCTCTTCCGATCT-3′ and the 5′-GGAGTTCAGACGTGTGCTCTTCCGATCT-3′ tails, respectively. PCRs were performed with Moltaq 16S (Molzym Life Science, Bremen, Germany) using the manufacturer’s protocol and 2 µL of microbial DNA. The cycling conditions are described in Poirier et al. [8]. All PCRs were performed in triplicate. Replicates were pooled and the amplified DNA was purified with a QIAquick kit (Qiagen, Hilden, Germany).

Sample multiplexing was performed by adding tailor-made 6-bp unique index tags to the ends of the forward and reverse adapters (5′-AATGATACGGCGACCACCGAGATCTACACT-3′ and 5′-CAAGCAGAAGACGGCATACGAGAT-NNNNNN-GTGACT-3′, respectively) on 50–200 ng of purified amplicons from the first PCR using 2.5 U of a DNA-free Taq DNA Polymerase and 1xTaq DNA polymerase buffer as detailed in Poirier et al. [8]. Amplicons were purified using CleanPCR magnetic beads (CleanNA, Alphen aan de Rijn, The Netherlands). The concentration of the purified amplicons was measured using a Nanodrop spectrophotometer (Thermo Scientific, Waltham, USA). All libraries were pooled using equal amounts. The pool was denatured (NaOH 0.1 N) and diluted to 7 pM. PhiX Control v3 (Illumina, San Diego, USA) was added to the pool at 4.5% of the final concentration. A volume of 600 μL was loaded onto the Illumina MiSeq cartridge according to the manufacturer’s instructions using the MiSeq Reagent Kit v3 (2 × 300 bp paired-end reads, 15 Gb output).

The quality of the sequencing was first evaluated using FastQC [9, 10]. The paired-end sequences were merged into contigs with PEAR v0.9.10 [11]. Adapters were trimmed with cutadapt v1.12 [12]. Low-quality bases at the extremities of sequences were removed using Sickle v1.330 [13]. Data were subsequently imported into the FROGS (Find Rapidly OTUs with Galaxy Solution) pipeline [14]. Sequences were dereplicated before being clustered using SWARM [15] with a local clustering threshold with a distance of 3. Chimeras were removed with vsearch [16]. OTUs only appearing more than 10 times in the whole dataset were kept [17]. Taxonomic assignment was performed using Silva 128 SSU [18] as reference database using in both cases the Blastn+ algorithm [19].

### Results

We first carried out a comparative alignment of both primers sequences to 16S rRNA encoding gene sequences of about 450 strains commonly found in food microbiota (data not shown). This alignment revealed that the mismatch differentiating both primers could have a specific impact on the amplification of *Leuconostoc, Oenococcus* and *Fructobacillus* genera. As shown in Table 1, we noticed while Muyzer-Primer1 has an A nucleotide on the fifth position from the 3′-end similarly to sequences belonging to *Weissella* genus. This A nucleotide being highly conserved in bacterial 16S rDNA, in particular within species belonging to *Firmicutes*. However, all other sequences of the three remaining genera of *Leuconostoceae* have a T nucleotide. By contrast, Klindworth-Primer2 sequence, which has a degenerated base W (A/T) at this position, should not induce any bias during the amplification of sequences belonging to all Leuconostocaceae.

**Table 1:** Sequence alignments of both V3 universal primers with the corresponding region of *Fructobacillus, Leuconostoc, Oenococcus* and *Weissella* species 16S rDNA encoding gene

Bacterial species	Sequences (5′–3′)
*Fructobacillus durionis*	ACGGGAGGC**T**GCAG
*Fructobacillus ficulneus*	ACGGGAGGC**T**GCAG
*Fructobacillus fructosus*	ACGGGAGGC**T**GCAG
*Fructobacillus pseudoficulneus*	ACGGGAGGC**T**GCAG
*Fructobacillus tropaeoli*	ACGGGAGGC**T**GCAG
*Leuconostoc gelidum* subsp. *gelidum*	ACGGGAGGC**T**GCAG
*Leuconostoc gelidum* subsp. *gasicomitatum*	ACGGGAGGC**T**GCAG
*Leuconostoc citreum*	ACGGGAGGC**T**GCAG
*Leuconostoc pseudomesenteroides*	ACGGGAGGC**T**GCAG
*Leuconostoc carnosum*	ACGGGAGGC**T**GCAG
*Leuconostoc mesenteroides*	ACGGGAGGC**T**GCAG
*Oenococcus alcoholitolerans*	ACGGGAGGC**T**GCAG
*Oenococcus kitaharae*	ACGGGAGGC**T**GCAG
*Oenococcus oeni*	ACGGGAGGC**T**GCAG
*Weissella ceti*	ACGGGAGGC**A**GCAG
*Weissella confusa*	ACGGGAGGC**A**GCAG
*Weissella cibaria*	ACGGGAGGC**A**GCAG
*Weissella hellenica*	ACGGGAGGC**A**GCAG
*Weissella viridescens*	ACGGGAGGC**A**GCAG
Muyzer-Primer1	ACGGRAGGC**A**GCAG
Klindworth-Primer2	ACGGRAGGC**W**GCAG

**Fig. 1:**
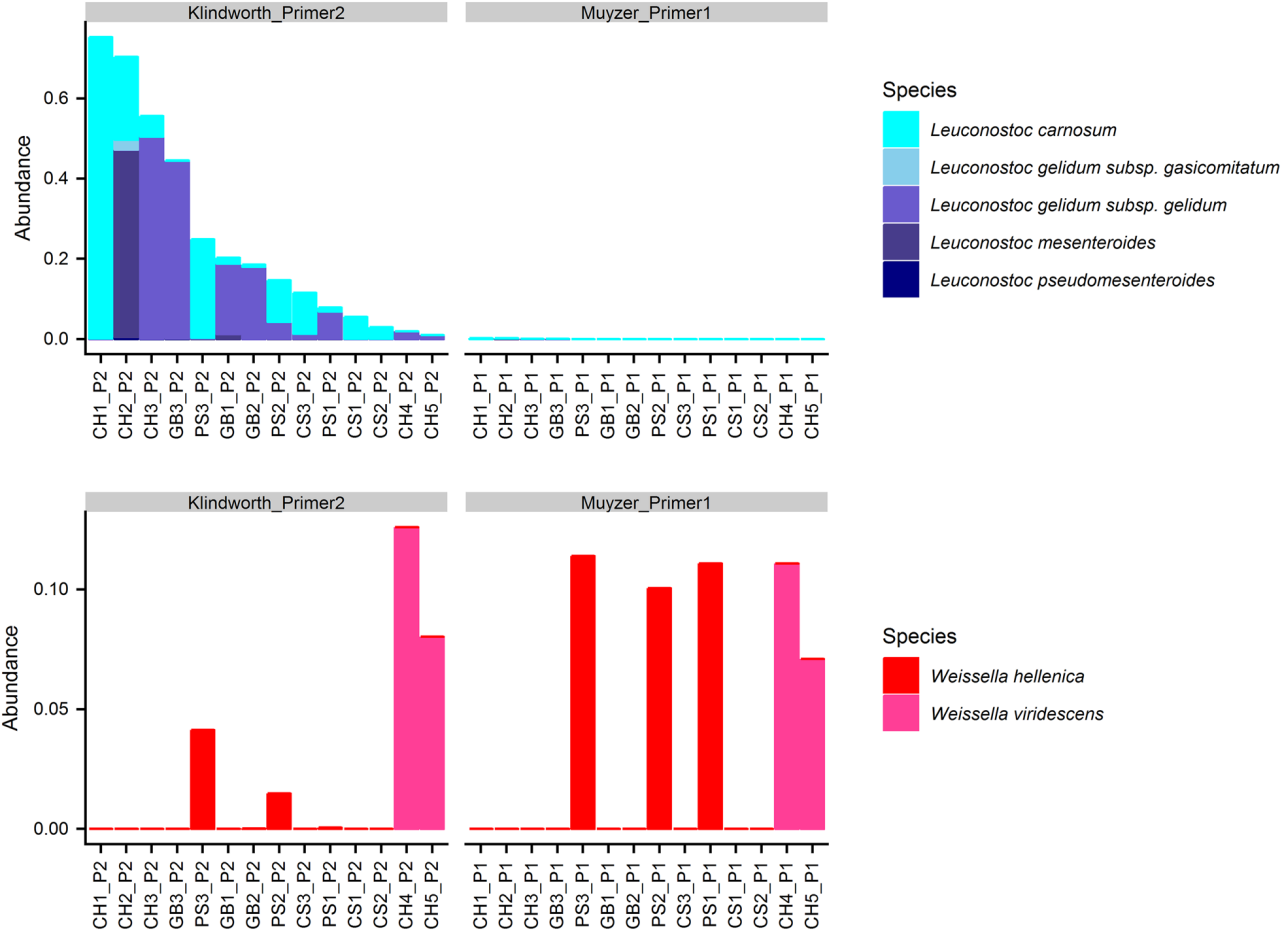
Composition plots of relative abundances of OTUs generated by 16S rRNA (V3–V4) sequencing within Leuconostocaceae at the genus level. **a** *Leuconostoc* species, **b** *Weissella* species. Results obtained with Klindworth-Primer2 are on the left side, those obtained with Muyzer-Primer1 are on the right side. The amplification bias is affecting all species of *Leuconostoc* tested

The relative bacterial composition within Leuconostocaceae of our chosen food samples and mock communities is detailed in Fig. 1. Less than 0.5% of sequence of *Leuconostoc* was detected within the samples amplified with Muyzer-Primer1. By contrast, proportions of OTUs assigned to *Leuconostoc* varied from 3% in CS2 up to 44% in GB3 within samples amplified with Klindworth-Primer2. This result clearly highlights that the use of Muyzer-Primer1 to monitor bacterial communities induces a serious bias for samples that contain *Leuconostoc* species. Moreover, results also showed that sequences of *Weissella* were amplified by both primers. However, the use of Muyzer-Primer1 leads to an overestimation of the relative abundance of this genus probably due to the non-amplification of *Leuconostoc*. This result was also confirmed by the 16S rRNA gene (region V3–V4) sequencing of mock communities. While no sequence of *Leuconostoc* was detected in both mock communities with Muyzer-primer1, Klindworth-primer2 allowed the amplification of 1.9% and 1% of sequences assigned to *Leuconostoc gelidum* subsp*. gelidum* in MC1 and MC2, respectively.

### Discussion

Exploring the diversity of microorganisms in foodstuffs has become a broad focus of research in food microbiology. However, the recent and rapid adoption of new generation sequencing techniques caused a certain backlog in proper evaluation of the primers used for diversity surveys. Notably, the percentage coverage and application scope of the primers used in previous studies are largely unknown.

Our study shows that even commonly used single primers only differentiated by a single mismatch could exhibit significant differences in the sequencing results. In some samples, 44% of the total microbial community carried by *Leuconostoc* species was missed.

Regarding the bibliography, we pointed out that this unappropriated Muyzer-Primer1 was still used in recent studies to describe food products, a situation that is somewhat problematic since these products may contain *Leuconostoc* in particular. For instance, Zhang et al. did not detect *Leuconostoc* in Yucha samples (a fermented food made with cooked rice and fresh fish) [6] while it is regularly found in this type of food product [20, 21]. Similarly, Pimentel et al. used Muyzer-Primer1 to characterize samples collected from cultured seabass *Dicentrarchus labrax* without detecting OTUs for this genus [22] whereas *Leuconostoc* are common on seafood products [23]. Ercolini et al. as well as Aponte et al. also failed to detect *Leuconostoc* with this primer whereas they could observe its presence in the same cheeses by targeting V4–V5 or V6–V8 regions [4, 5]. Moreover, the Muyzer-Primer1 was broadcast even recently by in silico studies promoting the use of this sequence [24, 25].

Despite its high coverage rate of 98% highlighted by Wang et al. [24], Muyzer-Primer1 cannot be suitable to characterize certain ecosystems and notably food products. Indeed, *Leuconostoc, Oenococcus* and *Fructobacillus* species play a relevant role either in food spoilage or in food and beverage fermentation in which they are of interest as a starter culture [26]. For instance, *Oenococcus oeni* is responsible for malolactic fermentation in wine, *Leuconostoc mesenteroides* is responsible for acetoin and butanediol production in cheese through citrolactic fermentation, whereas *Leuconostoc gelidum* pyruvate metabolism would trigger spoilage of fresh meat. Their good estimation within food microbiota is therefore crucial for understanding how food communities function during meat, cheese or wine fermentation but also for elucidating whether there is any possibility to develop new strategies to interfere with the growth and to postpone spoilage of packaged and refrigerated foods products. Furthermore, they are also widespread in the environment, and have been isolated from plant matter [27] and human clinical sources [28]. Consequently, in order to avoid accumulative bias and questionable biological conclusions, we recommend abandoning the use of this primer.

## Limitations (bullet points)

Although the number of samples tested in this study were few, our result was confirmed by the analysis of microbial community from various food products (cheese, cooked ham, cod fillet and salmon fillet), which were amplified with Muyzer-Primer1. Although these samples were not sequenced with Klindworth-Primer2 as a control, they revealed to be deprived of *Leuconostoc* species suggesting the general failure of Muyzer-Primer1 to amplify this genus. Furthermore, our study focused on a comparative analysis between *Leuconostoc* and *Weissella* but not on *Oenococcus* and *Fructobacillus*. However, it is very likely that these genera might not be amplified with Muyzer-Primer1.
